# A phase 1b study of erlotinib and momelotinib for the treatment of *EGFR*-mutated, tyrosine kinase inhibitor-naive metastatic non-small cell lung cancer

**DOI:** 10.1007/s00280-021-04369-0

**Published:** 2021-11-13

**Authors:** Sukhmani K. Padda, Karen L. Reckamp, Marianna Koczywas, Joel W. Neal, Jun Kawashima, Shengchun Kong, Daniel B. Huang, Mark Kowalski, Heather A. Wakelee

**Affiliations:** 1 Stanford University School of Medicine/Stanford Cancer Institute, Stanford, CA USA; 2 Present Address: Cedars-Sinai Medical Center, 8700 Beverly Blvd, SCCT 1S31, Los Angeles, CA 90048 USA; 3 City of Hope Comprehensive Cancer Center, Duarte, CA USA; 4 Gilead Sciences, Inc., Foster City, CA USA; 5 The Oncology Institute of Hope and Innovation, Santa Ana, CA USA; 6 Present Address: Sierra Oncology, Inc., Vancouver, BC Canada; 7 Present Address: Genentech, Inc., South San Francisco, CA USA

**Keywords:** JAK1/2, TBK1, EGFR, Lung cancer, Erlotinib

## Abstract

**Introduction:**

Preclinical evidence suggests the feedforward cytokine loop of interleukin-6/Janus kinases (JAK)/STAT3 plays a role in epidermal growth factor receptor tyrosine kinase inhibitor (EGFR TKI) resistance in *EGFR*-mutated non-small cell lung cancer (NSCLC).

**Methods:**

In this phase 1b study, the JAK1/2 and TANK-binding kinase 1 (TBK1) inhibitor momelotinib was evaluated in combination with erlotinib in patients with EGFR TKI-naive, *EGFR*-mutated NSCLC. After erlotinib lead-in (50, 75, 100, or 150 mg oral daily [QD]), momelotinib was combined and dose escalated in a 3 + 3 study design. The primary endpoint of maximum tolerated dose (MTD) of momelotinib was determined based on the incidence of dose-limiting toxicities (DLTs) during the first 28-day cycle. Secondary endpoints included efficacy and pharmacokinetics (PK).

**Results:**

Eleven patients were enrolled across 3 dose levels of momelotinib (100 mg QD, 200 mg QD, and 100 mg twice daily [BID]). The MTD was momelotinib 200 mg QD in combination with erlotinib. Two DLTs of grade 4 neutropenia without fever and grade 3 diarrhea occurred at momelotinib 100 mg BID. Most common treatment-emergent adverse events included diarrhea, dry skin, fatigue, and decreased appetite; the vast majority being grades 1–2. The overall response rate was 54.5% (90% CI 27.1–80.0; all partial) and median progression-free survival was 9.2 months (90% CI 6.2–12.4). Momelotinib did not affect the PK of erlotinib.

**Conclusions:**

The JAK1/2 and TBK1 inhibitor momelotinib in combination with erlotinib did not appear to enhance benefit over the historical data of erlotinib monotherapy in patients with *EGFR*-mutated NSCLC.

**ClinicalTrials.gov identifier:**

NCT02206763.

**Supplementary Information:**

The online version contains supplementary material available at 10.1007/s00280-021-04369-0.

## Introduction

The upfront treatment of advanced epidermal growth factor receptor (*EGFR*)-mutated non-small cell lung cancer (NSCLC) is rapidly changing. First- (e.g., erlotinib, gefitinib) and second-generation (e.g., afatinib, dacomitinib) EGFR tyrosine kinase inhibitors (TKIs) were routinely used in the frontline setting until the development of third-generation TKI osimertinib [1–5]. Despite osimertinib’s ability to inhibit the most common *EGFR*-sensitizing mutations and the acquired resistance mutation T790M, resistance remains a problem [6]. There are two main types of resistance to EGFR TKI therapy, including on-target resistance and bypass mechanisms. With the first- and second-generation TKIs, the on-target resistance gatekeeper mutation T790M developed in approximately 60% of tumors at the time of progression [7, 8]. However, with third-generation TKI osimertinib, the complexity and heterogeneity of resistance mechanisms has increased when this therapy is used in the first- or second-line setting (i.e., in the context of T790M). On-target resistance occurs less frequently with osimertinib, with the *EGFR* C797S mutation developing in ~ 15%, whereas bypass tracts are more common, including amplification of *MET*, *HER2*, and *PIK3CA*, acquired mutations in the mitogen-activated protein kinase (MAPK)/phosphoinositide 3-kinase (PI3K) pathway and rare oncogenic fusions [9, 10]. Therefore, strategies to prevent and overcome resistance continue to remain important.

The interleukin-6 (IL-6)/Janus kinase (JAK)/signal transducer and activator of transcription 3 (STAT3) signaling pathway is overactive in multiple cancer types, including lung cancer, and is important in cancer pathogenesis [11]. IL-6 is the primary driver of this pathway, engaging with the transmembrane IL-6 receptor subunit-β (gp130). This leads to activation of gp130-associated JAK tyrosine kinases (JAK1, JAK2, and TYK2) and subsequent phosphorylation of tyrosine residues of gp130, which serve as docking sites for proteins that activate PI3K/AKT, MAPK, and JAK/STAT3 pathways. In addition, IL-6’s downstream activation of STAT3 induces IL-6 expression, resulting in a feedforward autocrine loop [12]. This coordinated interaction of pathways results in a hostile tumor microenvironment (e.g., promotion of dysfunctional angiogenesis, infiltration of fibroblasts, and recruitment of myeloid suppressor cells) and promotes cancer cell proliferation, survival, invasion, and metastatic potential [12, 13].

The IL-6/JAK/STAT3 pathway has been observed to be hyperactivated in *EGFR-*mutated NSCLC [14–17] and may play a role in resistance to EGFR TKI therapy [18, 19]. The potential therapeutic role of JAK inhibition for *EGFR-*mutated NSCLC has been known for some time. For example, in multiple *EGFR*-mutated NSCLC lines, Gao et al. observed excessive levels of IL-6 in cell culture and high levels of phosphorylated-STAT3 (pSTAT3). Pan-JAK inhibition (with P6) uniformly abrogated pSTAT3, whereas EGFR inhibition did not, resulting in cell-cycle arrest at the G_2_/M phase and suppressed colony formation [14]. The importance of abrogating STAT3 via inhibition of JAK kinases (e.g., observed with JAK2 inhibitor AZD1480 and JAK1 inhibitor CJ14939) has been demonstrated in other studies [15, 18, 20, 21]. Surprisingly, it has been observed that EGFR TKIs (e.g., erlotinib) induce feedback activation of STAT3 signaling in *EGFR*-mutant cell lines via autocrine- and paracrine-secreted factors such as IL-6, leading to increased numbers of resistant cells [19, 22].

Inhibition of the JAK/STAT pathway has demonstrated activity in EGFR TKI-sensitive *EGFR*-mutant models. Murakami et al. demonstrated that JAK2 inhibition (AZD1480) had independent activity in PC-9 xenograft tumors and *EGFR-*mutant transgenic mice, where treatment resulted in a decreased number of lung tumors and improved overall survival [20]. Looyenga et al. also demonstrated that the JAK1/2 inhibitor (ruxolitinib) decreased colony formation in soft agar assays and slowed tumor growth in HCC-827 xenograft models [21]. The combination of erlotinib plus JAK1/2 inhibition (ruxolitinib) was noted to have synergistic activity compared to EGFR inhibition alone in an HCC4006 xenograft model [19]. The combination of osimertinib and JAK1 inhibitor (AZD4205) had synergistic activity compared to each agent alone in PC-9 and HCC827 models, with an observed increased degree of tumor regression, delayed tumor regrowth, and evidence of knockdown of pSTAT3 [23, 24]. In addition to JAK1 inhibition (itacitinib) having independent activity in HCC827 xenograft model, synergistic tumor growth inhibition was also observed in combination with erlotinib or osimertinib [25]. JAK/STAT inhibition may also have a role in EGFR TKI-resistant models in combination with first-, second-, and third-generation TKIs [18, 22, 25–27].

Given the hyperactivation of the IL-6/JAK/STAT3 pathway in *EGFR*-mutated NSCLC, feedforward activation of the IL-6/JAK/STAT3 pathway in the presence of EGFR TKIs, and synergistic activity of JAK inhibition with EGFR TKIs in *EGFR*-mutant models, we conducted a phase 1b study of the combination of first-generation EGFR TKI erlotinib plus the JAK1/2 and TANK-binding kinase 1 (TBK1) inhibitor momelotinib in patients with EGFR TKI-naive, *EGFR*-mutated advanced NSCLC.

## Patients and methods

### Patients

Eligible patients had a pathologically confirmed diagnosis of metastatic NSCLC with a documented *EGFR* exon 19 deletion or exon 21 L858R substitution mutation. Patients were EGFR TKI-treatment–naive, except to erlotinib, in which stable doses for ≥ 11 days and ≤ 45 days were required; had an Eastern Cooperative Oncology Group performance status of 0, 1, or 2; and had measurable disease by Response Evaluation Criteria in Solid Tumors (RECIST) version 1.1 criteria [28]. Patients with treated or asymptomatic untreated brain metastases were eligible, whereas patients with active infection or carriers of hepatitis B or C were ineligible.

### Study design

The study was conducted at three sites in the United States from October 2014 through January 2017. The study was conducted in accordance with the Declaration of Helsinki and Good Clinical Practice guidelines. The study protocol was approved by an institutional review board at each site before enrollment of a patient. All patients provided written informed consent.

This was an open-label, dose-escalation phase 1b study, which comprised a dose-escalation phase, followed by a planned, randomized, expansion phase of the combination of erlotinib plus momelotinib vs. erlotinib alone (ClinicalTrials.gov Identifier: NCT02206763). An erlotinib lead-in was utilized during the dose-escalation phase since erlotinib’s toxicities of rash and diarrhea onset in the first 2–3 weeks of treatment [29] allowing for (i) dose adjustment for erlotinib-related toxicities as per clinical standard of care prior to combination with momelotinib; (ii) examination of the impact of momelotinib on erlotinib steady-state pharmacokinetics; and (iii) accurate assessment of toxicity attribution during the combination treatment period. However, the study was discontinued prior to the expansion phase due to the preliminary efficacy data from the dose-escalation phase and the changing treatment landscape of *EGFR*-mutated NSCLC.

Patients were screened within 28 days of the start of study treatment and cycles were defined as 28 days. Erlotinib was self-administered orally once daily (QD) at least 1 h before or 2 h after a meal, with doses permitted at 50, 75, 100, or 150 mg. Dose reductions were permitted for management of erlotinib-related toxicity such as rash and diarrhea. Momelotinib was dose escalated following a standard 3 + 3 design starting at 100 mg QD (Table 1). Momelotinib was self-administered orally QD or twice daily (BID) (Table 1), with the latter planned dosing frequency differing from that used in myelofibrosis trials [30]. Study treatment continued until disease progression, unacceptable toxicity, or consent withdrawal.

**Table 1:** Planned momelotinib dose-escalation cohorts

Dose levels	Momelotinib (oral)	Erlotinib (oral)
1	100 mg QD	150 mg QD^b^
2A^a^	200 mg QD	
2B^a^	100 mg BID	
3	150 mg BID	
4	200 mg BID	

*AEs* adverse events, *BID* twice daily, *DLT* dose-limiting toxicity, *QD* once daily

^a^Patients alternated in enrolling in level 2A and 2B, starting first with level 2A. If a DLT occurred in only 1 patient, only that dose level (2A or 2B) was expanded to 6 patients. Two DLTs occurred at dose level 2B. Cohorts 3 and 4 were not enrolled

^b^Doses permitted at 50, 75, 100, or 150 mg. Dose interruption and/or reduction were permitted for erlotinib-specific AEs, including during the DLT period

### Safety

The primary endpoint was the incidence of dose-limiting toxicities (DLTs). Physical exams and safety labs were performed on day 1 of cycle 1, day 15 of cycle 1, and on the first day of each cycle thereafter. Adverse events (AEs) were coded using the Medical Dictionary for Regulatory Activities (MedDRA), graded according to the Common Terminology Criteria for Adverse Events (CTCAE) version 4.03, and collected until 30 days after the last dose of the study treatment.

DLTs were assessed during the first 28 days of combined erlotinib and momelotinib treatment and defined as clinically significant AEs related to study treatment. Prespecified hematologic DLTs only included grade 4 neutropenia (absolute neutrophil count [ANC] < 500/μL) and grade 4 thrombocytopenia (platelet count < 25,000/μL). Important exceptions for related grade 3 nonhematologic toxicities included grade 3 nausea or vomiting ≤ 48 h duration, diarrhea or rash that improved to grade 0 to 1 within 21 days of interruption of erlotinib, and alanine aminotransferase (ALT) or aspartate aminotransferase (AST) ≥ 5 times the upper limit of normal (ULN) that improved to ≤ 3 times the ULN within 21 days. Treatment delays of ≥ 28 days due to a treatment-emergent AEs (TEAEs) were also considered DLTs. In addition, the treating investigators could deem a TEAE a DLT if in their opinion the TEAE was of potential clinical significance such that further dose escalation would have exposed patients to an unacceptable risk.

### Efficacy

Imaging by computed tomography (CT) with contrast was performed at baseline, every 8 weeks during year 1 and every 12 weeks thereafter. If a patient had brain metastases, brain magnetic resonance imaging (MRI) was repeated on the same schedule. Secondary endpoints included overall survival (OS), progression-free survival (PFS), and overall response rate (ORR) (i.e., proportion of complete and partial response) as assessed per RECIST version 1.1 [28].

### Pharmacokinetics

Prior to momelotinib dosing, blood samples were collected before a dose of erlotinib and at 0.5, 1, 2, 4, 6, 8, and 24 h after erlotinib dosing. On day 15 of cycle 1, blood samples were collected at the same time points after dosing with momelotinib and erlotinib (the 24-h time point was only applicable for momelotinib QD dosing). The concentrations of momelotinib and its major metabolite GS-644603 were evaluated using a liquid chromatography-tandem mass spectrometry (LC–MS/MS) assay with solid phase extraction validated over the range of 0.5–1000 ng/mL for each analyte. The concentration of erlotinib was determined using an LC–MS/MS assay with protein precipitation validated over the range of 5–5000 ng/mL. Both assays demonstrated requisite accuracy (± 15%) and precision (coefficient of variation [CV] < 15%). Pharmacokinetic (PK) parameters were estimated using standard noncompartmental methods with Phoenix WinNonlin^®^ software (Certara, Princeton, NJ).

### Statistical analysis

Any patient who received ≥ 1 dose of study drug was included in the safety and efficacy analyses. Descriptive statistics were used to summarize study characteristics, DLTs, and AEs, including by dose level. ORR was summarized with corresponding two-sided 90% exact confidence intervals (CIs) using the binomial distribution. Both OS and PFS were summarized using Kaplan–Meier estimates and corresponding 90% CIs. Patient concentration data and PK parameters for erlotinib, momelotinib, and its major metabolite GS-644603 were summarized by dose level using descriptive statistics.

## Results

### Patient characteristics

A total of 11 patients with treatment-naive, *EGFR*-mutated NSCLC were enrolled (Table 2), including 6 with L858R and 5 with exon 19 deletion. The median age was 55 years, the majority were female (*n* = 7) and of Asian and White race (*n* = 4 and *n* = 6, respectively). The median time since diagnosis of NSCLC was 1.8 months (range 1.3–2.9), as patients were permitted to be on erlotinib for up to 45 days prior to enrolling on the study. Two patients had undergone prior palliative radiotherapy to bone metastases.

**Table 2:** Baseline characteristics

	Dose level 1 Momelotinib 100 mg QD	Dose level 2A Momelotinib 200 mg QD	Dose level 2B Momelotinib 100 mg BID	Total
Number	3	3	5	11
Age (years)				
Median	57	65	52	55
Range	50–61	52–81	48–68	48–81
Female, *n* (%)	2 (66.7)	2 (66.7)	3 (60.0)	7 (63.6)
Race, *n* (%)				
Asian	2 (66.7)	1 (33.3)	1 (20.0)	4 (36.4)
White	1 (33.3)	2 (66.7)	3 (60.0)	6 (54.5)
Other	0	0	1 (20.0)	1 (9.1)
Ethnicity, *n* (%)				
Hispanic/Latino	0	1 (33.3)	0	1 (9.1)
Not Hispanic/Latino	3 (100.0)	2 (66.7)	5 (100.0)	10 (90.9)
ECOG PS, *n* (%)				
0	3 (100.0)	1 (33.3)	3 (60.0)	7 (63.6)
1	0	2 (66.7)	2 (40.0)	4 (36.4)
Current smoker, *n* (%)	0	0	0	0
Time since diagnosis, months				
Median	2.2	1.8	1.4	1.8
Range	1.7–2.9	1.8–2.7	1.3–1.8	1.3–2.9
Prior palliative radiation, *n* (%)	0	0	2 (40.0)	2 (18.2)

*BID* twice daily, *ECOG PS* European Cooperative Oncology Group performance status, *QD* once daily

### Exposure and disposition

The median duration of treatment exposure to momelotinib was 40.1 weeks (range 2.4–63.1) with a median number of 10 cycles received (range 0.6–15.8). The median duration of exposure to erlotinib was similar at 40.3 weeks (range 2.6–66.3) with a median of 10.1 cycles received (range 0.6–16.6). As a result of dose reductions (*n* = 2) and interruptions (*n* = 5), the average daily dose of momelotinib among patients enrolled at dose level 2A (200 mg QD) and 2B (100 mg BID) was lower than the assigned dose: 178.2 ± 27.1 mg and 186.7 ± 26.5 mg, respectively. The 2 dose reductions of momelotinib were due to AEs (1 each in dose level 2A and 2B). All-cause AEs leading to dose interruption or modification of momelotinib were diarrhea (*n* = 2 patients), pericardial effusion (*n* = 1), dyspnea (*n* = 1), acute myocardial infarction (*n* = 1), pneumonitis (*n* = 1), and myalgia, abdominal discomfort, and fatigue (*n* = 1). All patients were taking erlotinib 150 mg QD at the start of cycle 1, except 1 patient on erlotinib 100 mg QD in dose level 1. There were also dose reductions of erlotinib performed in 4 patients (*n* = 4), across all dose levels and all for AEs, including 1 to 50 mg.

Momelotinib was discontinued for progressive disease (PD) in 7 (63.6%) patients, AEs in 3 (27.3%) patients (1 each of grade 3 follicular rash, grade 1 hepatitis B, and grade 4 neutropenia), and per patient discretion in 1 (9.1%) patient. Erlotinib was discontinued due to PD in 7 (63.6%) patients, AEs in 2 (18.2%) patients (1 each of grade 4 neutropenia and grade 1 hepatitis B), and per investigator or patient decision in 1 patient each.

### Safety

DLTs were observed in 2 of 5 patients at dose level 2B (momelotinib 100 mg BID). The DLTs were related to both erlotinib and momelotinib. One patient had a DLT of grade 4 neutropenia without fever at day 16 that resolved without treatment on day 19 but resulted in permanent discontinuation of both drugs. The second patient had a DLT of grade 3 diarrhea on day 15 that resolved on day 22, resulting in a momelotinib dose reduction to 150 mg QD with no change in erlotinib dose (150 mg QD). Although there was a DLT exception for grade 3 diarrhea, in light of this toxicity leading to a dose reduction of momelotinib and the other toxicity observed in this cohort, this TEAE was labeled as a DLT due to its clinical significance based on the opinion of the investigators that further dose escalation would have exposed patients to unacceptable risk. There was no DLT observed at dose level 1A (momelotinib 100 mg QD) or dose level 2A (momelotinib 200 mg QD). Based on these findings, the maximum tolerated dose (MTD) was momelotinib 200 mg QD in combination with a standard dosing of erlotinib (i.e., dose level 2A; *n* = 3). All patients were taking erlotinib 150 mg QD in dose level 2A at the start of cycle 1, with 1 patient requiring a dose reduction to 100 mg QD on day 14 due to an erlotinib-related toxicity.

All 11 patients had at least 1 AE. A total of 7 patients (63.6%) had an AE of grade 3 severity or greater, including 3 patients (100%) at dose level 2A and 4 patients (80%) at dose level 2B. There were serious AEs (SAEs) observed in 3 patients (27.3%), including 2 patients in dose level 2A and 1 patient in dose level 2B. Of note, there was no AE of grade 3 severity or greater or SAE observed in dose level 1. There were no treatment-related deaths observed in the study.

The most common TEAEs of the combination included diarrhea, dry skin, fatigue, and decreased appetite (Table 3). The most common AEs that were considered related to momelotinib included diarrhea (54.5%), nausea (36.4%), fatigue (36.4%), dysgeusia (27.3%), and neutropenia (27.3%). The most common AEs that were considered related to erlotinib included diarrhea (63.6%) and skin-related toxicities (i.e., 54.5% each of dry skin, paronychia, and rash).

**Table 3:** TEAEs reported in > 20% of patients of any grade

TEAEs^a^	Dose level 1 Momelotinib 100 mg QD *n* = 3 *n* (%)	Dose level 2A Momelotinib 200 mg QD *n* = 3 *n* (%)	Dose level 2B Momelotinib 100 mg BID *n* = 5 *n* (%)	Total Any grade *n* = 11 *n* (%)	Total Grades 3–4^b^ *n* = 11 *n* (%)
Diarrhea	1 (33.3)	3 (100.0)	3 (60.0)	7 (63.6)	1 (9.1)^c^
Dry skin	2 (66.7)	2 (66.7)	3 (60.0)	7 (63.6)	
Fatigue	2 (66.7)	2 (66.7)	3 (60.0)	7 (63.6)	
Decreased appetite	2 (66.7)	3 (100.0)	2 (40.0)	7 (63.6)	
Cough	2 (66.7)	1 (33.3)	3 (60.0)	6 (54.5)	
Paronychia	1 (33.3)	2 (66.7)	3 (60.0)	6 (54.5)	
Urinary tract infection	0	2 (66.7)	4 (80.0)	6 (54.5)	
Nausea	2 (66.7)	1 (33.3)	2 (40.0)	5 (45.5)	
Alopecia	1 (33.3)	2 (66.7)	2 (40.0)	5 (45.5)	
Rash	1 (33.3)	2 (66.7)	2 (40.0)	5 (45.5)	1 (9.1)^d^
Headache	2 (66.7)	1 (33.3)	2 (40.0)	5 (45.5)	
Abdominal pain	0	2 (66.7)	2 (40.0)	4 (36.4)	1 (9.1)
Gastroesophageal reflux disease	1 (33.3)	0	3 (60.0)	4 (36.4)	
Dyspnea	2 (66.7)	1 (33.3)	1 (20.0)	4 (36.4)	1 (9.1)
Epistaxis	2 (66.7)	1 (33.3)	1 (20.0)	4 (36.4)	
Nasal dryness	1 (33.3)	2 (66.7)	1 (20.0)	4 (36.4)	
Chest discomfort	1 (33.3)	2 (66.7)	1 (20.0)	4 (36.4)	
Upper respiratory tract infection	1 (33.3)	1 (33.3)	2 (40.0)	4 (36.4)	1 (9.1)^e^
Muscle spasms	0	2 (66.7)	2 (40.0)	4 (36.4)	
Dry eye	2 (66.7)	0	2 (40.0)	4 (36.4)	
Vision blurred	2 (66.7)	1 (33.3)	1 (20.0)	4 (36.4)	
Dysgeusia	0	2 (66.7)	2 (40.0)	4 (36.4)	
Constipation	0	1 (33.3)	2 (40.0)	3 (27.3)	1 (9.1)
Dry mouth	0	2 (66.7)	1 (20.0)	3 (27.3)	
Vomiting	1 (33.3)	1 (33.3)	1 (20.0)	3 (27.3)	
Erythema	1 (33.3)	1 (33.3)	1 (20.0)	3 (27.3)	
Hypertrichosis	1 (33.3)	1 (33.3)	1 (20.0)	3 (27.3)	
Skin fissures	1 (33.3)	0	2 (40.0)	3 (27.3)	
Hematuria	1 (33.3)	1 (33.3)	1 (20.0)	3 (27.3)	
Neutropenia	0	1 (33.3)	2 (40.0)	3 (27.3)	2 (18.1)^c^

*BID* twice daily, *DLT* dose-limiting toxicity, *QD* once daily

^a^Severity graded according to Common Terminology Criteria for Adverse Events version 4.03

^b^Only 2 grade 4 events (neutropenia and sepsis). There was 1 grade 3 pneumonitis in dose level 2B not listed in the table

^c^DLTs (1 grade 3 diarrhea, 1 grade 4 neutropenia)

^d^There was grade 3 follicular rash and grade 3 papular rash in 1 patient at dose level 2A

^e^There was a grade 3 upper respiratory infection co-occurring with grade 3 kidney infection and grade 4 sepsis in 1 patient at dose level 2A. Grade 3 pneumonia was also reported

Of note, a low neutrophil count was observed in 5 (45%) patients, including a grade 3 event occurring in a patient at dose level 2A on day 15 and a grade 4 event occurring in a patient at dose level 2B (previously described DLT); the remaining were grade 1 to 2 in severity and all in dose level 2B. The 3 that were coded as AEs per protocol-specified definition (i.e., requiring intervention or dose modification) were considered related to erlotinib and momelotinib, respectively. There were no other recurrent grade 3 or greater hematologic laboratory abnormalities observed. The majority of chemistry laboratory abnormalities were grades 1 to 2, with the most common being AST (72.7%) and ALT increase (54.5%).

There were no prespecified AEs of special interest defined in the protocol. However, the following were highlighted: grade 1 peripheral sensory neuropathy in 1 patient at day 142 in dose level 2B; a grade 1 reactivation of hepatitis B in 1 patient that occurred 1 year after start of study treatment, the latter resulting in discontinuation of momelotinib and erlotinib; and no event of cataracts was reported. There was also 1 case of grade 3 pneumonitis considered related to momelotinib at dose level 2B.

### Efficacy

**Fig. 1:**
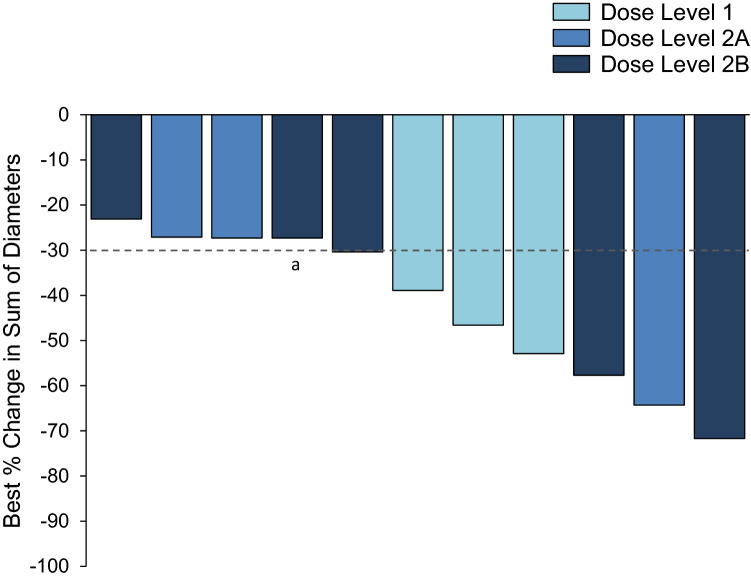
Best percentage change from baseline in tumor size by dose level (*N* = 11). ^a^Patient with best overall response of progressive disease

Confirmed objective responses (all partial) were observed in 6 of 11 patients for an ORR of 54.5% (90% CI 27.1–80.0). An additional 4 patients (36.4%) had stable disease and 1 had PD (9.1%; dose level 2B). Responses were observed in all dose levels. Across dose levels, the mean best percentage change in the sum of diameters of target lesions was −42.5 ± 17.1%, with reductions from baseline ranging from 23.1 to 71.7% (Fig. 1). The median duration of response (DoR) was 7.2 months (90% CI 4.4–9.6). The longest DoR was 11.2 months in a patient at dose level 2B.

At the date of data finalization on April 24, 2017, 10 of 11 patients had PD, and no death was reported. One patient enrolled at dose level 2A had discontinued the study without a documented event of PD or death. The median PFS was 9.2 months (90% CI 6.2–12.4). The median OS was not reached.

### Pharmacokinetics

The steady-state PK of momelotinib and its major metabolite GS-644603 was evaluated following administration of momelotinib in combination with erlotinib at day 15 of cycle 1. One patient in dose level 1 was receiving erlotinib 100 mg QD; all other patients in the PK analysis set were receiving erlotinib 150 mg dosing. Momelotinib peak concentration was reached approximately 1 to 2 h after dosing. Comparing momelotinib 200 mg QD to 100 mg QD, there was a slightly less than dose-proportional increase in maximum concentration (C_max_) and a slightly more than dose-proportional increase in exposure (AUC_tau_) (Table 4). Applying the same comparison to the metabolite GS-644603, there was also a slightly less than dose-proportional increase in C_max,_ while there was an approximately dose-proportional increase in AUC_tau_. The mean metabolite to parent ratios across dose levels ranged from 0.12 to 0.17 and 0.11 to 0.13 for AUC_tau_ and C_max_, respectively (Table 4).

**Table 4:** Pharmacokinetic parameters for momelotinib and its metabolite GS-644603

	Dose level 1 Momelotinib 100 mg QD *n* = 2	Dose level 2A Momelotinib 200 mg QD *n* = 2	Dose level 2B Momelotinib 100 mg BID *n* = 4^a^
Momelotinib			
C_max_, ng/mL	528.5 (54.7)	852.5 (22.1)	884.5 (14.8)
AUC_tau_, ng • mL/h	4000.4 (35.0)	9842.3 (31.0)	5219.0 (29.7)
T_max_, h	1.0 (1.0, 1.0)	1.5 (1.0, 2.0)	1.0 (1.0, 1.5)
* t*_1/2_, h	11.8 (9.1, 14.5)	10.2 (6.1, 14.3)	8.6 (5.4, 9.9)
GS-644603			
C_max_, ng/mL	65.7 (19.6)	92.7 (11.1)	95.1 (28.9)
AUC_tau_, ng • mL/h	685.0 (28.2)	1371.9 (25.0)	797.1 (27.6)
T_max_, h	1.5 (1.0, 2.0)	2.0 (2.0, 2.0)	2.0 (1.5, 2.0)
* t*_1/2_, h	27.7 (14.1, 41.3)	15.5 (8.3, 22.7)	8.4 (6.4, 10.8)
GS-644603:momelotinib ratio			
C_max_	0.13 (37.1)	0.11 (11.2)	0.11 (30.6)
AUC_tau_	0.17 (7.2)	0.14 (6.3)	0.12 (49.0)

Data for C_max_ and AUC_tau_ are presented as the mean (percent coefficient of variation); data for T_max_ and *t*_1/2_ are presented as median (Q1, Q3), and GS-644603/momelotinib ratios are presented as the mean (percent coefficient of variation)

*AUC*_*tau*_ area under the concentration versus time curve over the dosing interval, *BID* twice daily, *C*_*max*_ maximum concentration, *Q1/Q3* quartile 1/quartile 3, *QD* once daily, *t*_*1/2*_ half-life, *T*_*max*_ time to maximum concentration

*N* = 3 for AUC_tau_ for momelotinib, and *N* = 3 for AUC_tau_ and t_1/2_ for its metabolite

Erlotinib exposure as reflected in C_max_ and AUC_tau_ was comparable between monotherapy (i.e., day 1 of cycle 1) and combination therapy with momelotinib (day 15 of cycle 1) across all the momelotinib dose level groups. These data suggest there is no impact of momelotinib on the PK of erlotinib. (Supplementary Table 1).

## Discussion

We conducted a multisite, phase 1b, open-label study of the first-generation EGFR TKI erlotinib in combination with JAK1/2 and TBK1 inhibitor momelotinib in 11 patients with EGFR TKI-treatment–naive *EGFR*-mutated advanced NSCLC. The MTD was momelotinib 200 mg QD in combination with standard dosing of erlotinib, with QD dosing of 50, 75, 100, and 150 mg permitted. Two DLTs were observed when momelotinib was dosed at 100 mg BID in combination with erlotinib, including grade 3 diarrhea and grade 4 neutropenia. The treatment-related toxicities observed were commonly noted for each agent alone, including diarrhea, nausea, and fatigue for momelotinib [30–32] and diarrhea and skin toxicities for erlotinib [2, 33, 34]. A high rate of decreased neutrophil count was observed, occurring in 5 of 11 patients, grades 1 to 2 in 3 patients, and grades 3 and 4 in 1 patient each. Three of these low neutrophil count events were considered AEs (i.e., requiring intervention or dose modification), including 1 also being a DLT per prespecified protocol definitions. None were associated with fever or infection, and all were considered related to both erlotinib and momelotinib. Only the grade 4 neutropenia DLT resulted in discontinuation of treatment with erlotinib and momelotinib. Neutropenia rates with erlotinib monotherapy in randomized phase 3 studies were reported in 0% [2], 4.5% (plus 1.8% decreased neutrophils) [33], and 6% [34]. However, ANCs less than < 1000/mm^3^ (i.e., grade 3 or greater) were rarely observed with erlotinib monotherapy [33, 34]. There was no neutropenia event with momelotinib dosed up to 150 mg BID as reported in combination with MEK1/2 inhibitor trametinib in patients with *KRAS*-mutated NSCLC [35].

Although there remains extensive preclinical promise of targeting the JAK/STAT pathway for *EGFR*-mutated NSCLC [19–21, 23–25], we observed that the preliminary efficacy of the combination of JAK1/2 inhibitor, momelotinib, and erlotinib was similar to erlotinib monotherapy, including an ORR of 54.5% (90% CI 27.1–80.0) and a median PFS of 9.2 months (90% CI 6.2–12.4) [2, 33, 34]. In prior studies with erlotinib, the ORR ranged from 62.7 to 83%, and the median PFS ranged from 9.7 to 11 months [2, 33, 34]. In addition, the toxicity observed in dose level 2B (100 mg BID) did not allow further dose escalation to the target dose of 200 mg BID. Erlotinib PK were not affected by momelotinib, whereas the momelotinib maximum concentration and exposure were higher than previously reported with a prolonged terminal elimination half-life [35]. In addition, C_max_ and AUC_tau_ of the major metabolite GS-644603 were several-fold lower than previously reported [35–38]. Erlotinib is a potent inhibitor of aldehyde oxidase [39], which is necessary for the formation of GS-644603 [40], perhaps accounting for the low levels of GS-644603 observed in this study. The augmented momelotinib exposure noted in this study may partly account for the higher rates of neutropenia observed compared with the combination study of momelotinib and trametinib in *KRAS*-mutated NSCLC [35]. In an exploratory analysis, after 2 weeks on treatment with momelotinib, pSTAT3 was measured in IL-6–stimulated lymphocytes using validated phospho-flow cytometry assay on the whole blood of 8 patients (data not shown). Momelotinib temporarily inhibited pSTAT3 shortly after dosing, but there was no correlation between the total plasma momelotinib concentration and pSTAT3 inhibition. Of note, in vitro studies suggest that the momelotinib metabolite GS-644603 is approximately three-fold less potent as an inhibitor of JAK1/2-mediated STAT3 phosphorylation in IL-6–stimulated human peripheral blood mononuclear cells with a mean half maximal effective concentration of 689 nM vs. 259 nM for momelotinib [40]. Unfortunately, no on-treatment biopsy samples were available to examine the suppression of the JAK-STAT pathway in the tumor microenvironment. Considering the preliminary efficacy seen in the phase 1 dose escalation and the changing treatment landscape of *EGFR*-mutated NSCLC, the decision was made to not proceed to the randomized dose expansion of this combination.

Although this study was initiated based on the hypothesis of JAK inhibition preventing resistance to EGFR TKI therapy, there have also been preclinical studies demonstrating the potential utility of combining EGFR TKI therapy and JAK1/2 inhibitors in resistant models [26]. These combinations either restored EGFR TKI sensitivity or resulted in synergy, as demonstrated in both *EGFR* T790M models [18, 26, 41] and models without T790M or *MET* amplification [22]. This was observed across first- [18, 22, 41], second- [26], and third-generation TKIs [25], and from in vitro cell line studies to in vivo xenograft studies [18, 22, 25, 26, 41]. Unfortunately, these promising findings did not translate into transformative clinical benefit for patients in the current studies conducted [42, 43]. In a combination study of erlotinib and ruxolitinib in patients with documented progression on erlotinib, 1 of 22 patients (5%) had an objective response and the median PFS was only 2.2 months (95% CI 1.5–4.1) [43]. The efficacy was only modestly better in a combination study of afatinib and ruxolitinib in patients who had progressed on at least 1 first-generation EGFR TKI [42]. Five of 20 patients (25%) with *EGFR* T790M had an objective response with a median PFS of 4.9 months (95% CI 2.5–7.3), while 2 of 10 patients (20%) without *EGFR* T790M had an objective response and median PFS of 3.1 months (95% CI 0.0–8.8). There are ongoing studies of third-generation EGFR TKI osimertinib in combination with JAK1 inhibitor itacitinib (NCT03450330 and NCT02917993) in EGFR TKI-resistant NSCLC.

Momelotinib also inhibits TBK1 [35, 44], which is critical for type I interferon (IFN) production (e.g., IFN-beta) in an autocrine loop and as part of broader antiviral signaling. The TBK1/IRF3/IFN pathway has been recently described as an important mechanism of acquired resistance to EGFR inhibition [45]. In *EGFR*-mutant preclinical models, EGFR inhibition led to feedforward activation of TBK1 and its transcription factor IRF3, which is responsible for type 1 IFN transcription. Targeting the TBK1/IRF3/IFN pathway enhanced sensitivity to erlotinib and afatinib in *EGFR*-mutant sensitive and T790M xenograft models, respectively. Targeting this pathway also restored sensitivity to erlotinib in non-T790M, non-*MET*–amplified, EGFR TKI-resistant cell lines. Due to momelotinib’s ability to target TBK1, it was studied in *KRAS*-mutated NSCLC in combination with the MEK 1/2 inhibitor trametinib [35] and in pancreatic adenocarcinoma in combination with nab-paclitaxel and gemcitabine chemotherapy [38]. However, adding momelotinib did not improve efficacy over the historical control trametinib monotherapy [35] and chemotherapy, respectively [38].

## Conclusion

The combination of the JAK1/2 and TBK1 inhibitor momelotinib with erlotinib did not enhance benefit over historical controls of erlotinib monotherapy in patients with *EGFR*-mutated NSCLC and did not support enrollment to the randomized dose expansion [2, 33, 34]. However, there is growing evidence that feedforward activation loops, including IL-6/JAK/STAT3, may be important in the development of EGFR TKI resistance in patients with *EGFR*-mutated NSCLC [19, 45, 46]. Feedback activation of STAT3 in oncogene-addicted cancers has also been demonstrated with other targeted therapies, implicating a potential role of targeting these pathways more broadly in the genomic subtypes of lung cancer [19].

## Supplementary Information

Below is the link to the electronic supplementary material.

Supplementary file1 (DOCX 33 KB)

## Data Availability

The datasets generated during and/or analyzed during the current study are available from Sierra Oncology, Inc. upon reasonable request. Interested parties should contact Mark Kowalski MD PhD, Chief of Research and Early Development at mkowalski@sierraoncology.com.
