# Supplementary material for: A phase 1b study of erlotinib and momelotinib for the treatment of EGFR-mutated, tyrosine kinase inhibitor-naive metastatic non-small cell lung cancer
Source: Cancer Chemother Pharmacol. 2021 Nov 13;89(1):105–15. doi: 10.1007/s00280-021-04369-0 (PMC8739290; doi:10.1007/s00280-021-04369-0)
Supplement: Supplementary file 1 — Supplementary file1 (DOCX 33 KB) [file 280_2021_4369_MOESM1_ESM.docx]

# Supplemental Materials to:

Padda SK,* Reckamp KL, Koczywas M, et al.

A Phase 1b Study of Erlotinib and Momelotinib for the Treatment of EGFR-Mutated, Tyrosine Kinase Inhibitor−Naive Metastatic Non-Small Cell Lung Cancer.

*Cancer Chemotherapy and Pharmacology.*

***Corresponding author**:

Sukhmani K. Padda

Stanford University School of Medicine/Stanford Cancer Institute, Stanford, CA, USA

E-mail: sukhmani.padda@cshs.org

Contents

[Supplementary Table 12](#SupplTable1)

**Supplementary Table 1.** **Pharmacokinetic Parameters for Erlotinib Assessed During Monotherapy and in Combination with Momelotinib**

|  | **Dose Level 1**  **Momelotinib 100 mg QD** | | **Dose Level 2A**  **Momelotinib 200 mg QD** | | **Dose Level 2B**  **Momelotinib 100 mg BID** | |
| --- | --- | --- | --- | --- | --- | --- |
|  | **Day 1**  **(n = 3)** | **Day 15**  **(n = 2)** | **Day 1**  **(n = 3)** | **Day 15**  **(n = 2)** | **Day 1**  **(n = 5)** | **Day 15**  **(n = 5)** |
| C_max_, ng/mL | 1810.0 (34.0) | 1405.0 (35.7) | 2040.0 (42.5) | 1740.0 (17.9) | 1899.2 (33.2) | 1782.0 (17.3) |
| AUC_tau_, ng • mL/h | 28,304.7^a^ (2.1) | 19,804.5 (36.6) | 36,629.3 (34.5) | 29,655.1 (19.7) | 29,938.9^b^ (29.3) | 35,006.3^c^ (26.5) |

All patients were on erlotinib 150 mg QD, except 1 patient in dose level 1 was on erlotinib 100 mg QD. Data for C_max_ and AUC_tau_ are presented as the mean (percent coefficient of variation).

*BID*  twice daily, *QD* once daily

^a^N = 2; ^b^N = 4; ^c^N = 3
